# IDENTIFYING AGE-FRIENDLY CAMPUS STRATEGIES USING A DELPHI PANEL METHODOLOGY

**DOI:** 10.1093/geroni/igad104.0644

**Published:** 2023-12-21

**Authors:** Joann Montepare

**Affiliations:** Lasell University, Newton, Massachusetts, United States

## Abstract

This presentation will describe the results of a Delphi panel study of 18 experts in higher education who independently assessed age-friendly strategies in three core areas of institutional function: teaching and learning, personnel practices, and student affairs. To begin, experts evaluated the relative importance of 21 common challenges for advancing age inclusivity on U.S. campuses. They then assessed the extent to which 53 potential strategies were appropriate, feasible, and likely to be implemented by campus leaders. In the first round of evaluations, 3 of the 21 challenges were rated as not very important by some experts. On the second round of evaluations, experts assessed why these particular challenges were rated as such. During the first round of reviews, experts also indicated that while 8 strategies were feasible and appropriate to addressing challenges to age inclusivity, they were unlikely to be implemented on campuses. Thus, in the second round, experts were asked why this might have been the case by selecting 2 probable barriers from a list of barriers to adoption. Finally, experts rated the potential impact of all strategies for improving age inclusivity. That is, aside from feasibility or likelihood, the extent to which they thought the strategies could transform age-inclusive environments in higher education. In addition to describing which strategies were most highly recommended, insights will be presented about how the experts viewed various age-friendly challenges and the barriers that hold institutions back from taking up more age-inclusive practices to support older students, faculty, and staff.

